# A Survey on Deep-Learning-Based LiDAR 3D Object Detection for Autonomous Driving

**DOI:** 10.3390/s22249577

**Published:** 2022-12-07

**Authors:** Simegnew Yihunie Alaba, John E. Ball

**Affiliations:** Department of Electrical and Computer Engineering, James Worth Bagley College of Engineering, Mississippi State University, Starkville, MS 39762, USA

**Keywords:** autonomous vehicles, classification, deep learning, deep learning for point cloud processing, LiDAR, sparsity, 3D object detection

## Abstract

LiDAR is a commonly used sensor for autonomous driving to make accurate, robust, and fast decision-making when driving. The sensor is used in the perception system, especially object detection, to understand the driving environment. Although 2D object detection has succeeded during the deep-learning era, the lack of depth information limits understanding of the driving environment and object location. Three-dimensional sensors, such as LiDAR, give 3D information about the surrounding environment, which is essential for a 3D perception system. Despite the attention of the computer vision community to 3D object detection due to multiple applications in robotics and autonomous driving, there are challenges, such as scale change, sparsity, uneven distribution of LiDAR data, and occlusions. Different representations of LiDAR data and methods to minimize the effect of the sparsity of LiDAR data have been proposed. This survey presents the LiDAR-based 3D object detection and feature-extraction techniques for LiDAR data. The 3D coordinate systems differ in camera and LiDAR-based datasets and methods. Therefore, the commonly used 3D coordinate systems are summarized. Then, state-of-the-art LiDAR-based 3D object-detection methods are reviewed with a selected comparison among methods.

## 1. Introduction

Autonomous driving has succeeded since the 2007 DARPA urban challenge [1]. It has a high potential for decreasing traffic congestion, improving overall driving and road safety, fast and effective decision-making, and reducing carbon emission [2]. Autonomous vehicles need to perceive, predict, plan, decide, and execute decisions in an uncontrolled complex real world to achieve these goals, which is challenging. A small mistake in understanding the environment, plan, or decision causes fatal effects. A robust autonomous system is needed to avoid such mistakes. One branch of an autonomous system, the perception system, especially 3D object detection, helps to understand the driving environments, such as other vehicles and pedestrians. For the robust operation of the next branches in the autonomous system, the perception system should give precise information about the driving environment. It should also be robust enough to work in bad weather such as rain, snow, and fog and make fast and effective decisions to match high-speed driving [2,3].

The 3D sensors, such as Light Detection and Ranging (LiDAR) and Radio Detection and Ranging (radar), provide 3D information about the environment, including distance and speed estimation. Other sensors, such as depth sensors (RGB-D cameras), can also provide 3D information [4]. However, environmental variations, such as inclement weather, sensor limitations, and resolution differences, limit sensor performance. The LiDAR sensor is more robust to adverse weather than the camera but poorer in color and texture information. A solid-state LiDAR called flash LiDAR [5] uses optical flash operation to give texture information such as a standard camera. The major limitation of LiDAR sensors are the high price and struggle to detect close- or far-distance objects [5]. LiDAR data are sparse and unstructured, which makes processing LiDAR data challenging. On the other hand, a radar sensor is more robust in bad weather, better for long-range detection, and relatively cheaper. However, it has a lower resolution.

Due to the sparsity and unstructured nature, it is challenging to process LiDAR data. Different methods have been developed to process LiDAR point cloud data: projecting to 2D space, such as [6] MV3D [7] and AVOD [8], voxels, such as VoxelNet [9], F-PointNet [10], MVFP [11], and raw point clouds, such as PointNet [12], PointNet++ [13], and PointRCNN [14]. The projection method converts the 3D LiDAR point cloud into a 2D plane representation, such as a range view or bird’s-eye view (BEV). The projected data can be processed using the standard 2D detection models and reduces the computational burden due to 3D convolution; however, there is an information loss converting the LiDAR point cloud into 2D space representations. The voxel method discretizes the sparse point cloud data into a volumetric 3D grid of voxels and uses a series of voxels to represent the 3D point cloud. This representation avoids the unstructured nature of point clouds, but the 3D convolution is still the bottleneck and causes information loss from converting one representation to another [3]. On the other hand, the raw point cloud representation directly processes point cloud data to keep the information. However, the sparsity of the point cloud data and the high computational cost because of 3D convolution are challenges to this method (see Section 4 for details). Different compression techniques have been developed to remove spatial and temporal redundancies of point cloud data, to reduce the large volume, such as [15,16,17]. Although these compression techniques reduce redundancies, they can affect the performance of models (Details of compression techniques are out of the scope of this survey).

The major contributions of the paper can be summarized as follows:

1. An in-depth analysis of LiDAR-based 3D object detection, state-of-the-art (SOTA) methods, and a comparison of SOTA methods are presented.
2. The LiDAR processing and feature-extraction techniques are summarized.
3. The 3D coordinate systems commonly used in 3D detection are presented.
4. We categorize deep-learning-based LiDAR 3D detection methods based on LiDAR data processing techniques as projection, voxel, and raw point cloud.

The rest of the paper is organized as follows. Section 2 presents related work. Feature-extraction methods, stages of autonomous driving, and 3D coordinate systems representation are provided in Section 3. Section 4 summarizes the LiDAR 3D detection methods and compares the selected ones. The summary of the survey paper is presented in Section 5.

## 2. Related Work

Due to the rapid growth of deep learning (DL) in computer vision, object detection has become an extensively studied application. Three-dimensional detection draws much attention in robotics applications, such as autonomous driving, because the intention to know object driving environment and location has increased. Most review papers present general detection in 2D and 3D and for multiple sensors, mostly cameras and LiDARs. This work comprehensively reviews LiDAR 3D object-detection models for autonomous driving.

General object detection and semantic segmentation in 2D and 3D were presented in [2]. The available datasets and methods for autonomous driving were reviewed. Jiao et al. [18] presented DL-based methods, sensors, and datasets. However, this work focused mostly on 2D detection. Arnold et al. [4] reviewed 3D detection methods for autonomous driving. Rahman et al. [19] also reviewed 3D methods, datasets, and open challenges in 3D detections. Similarly, Li et al. [20] and Fernandes et al. [21] presented detection and segmentation in autonomous driving using LiDAR. DL-based 3D detection and segmentation methods in autonomous driving were also presented in [22]. Qian et al. [23] published a 3D detection method for autonomous driving. Alaba et al. [3] presented multisensor fusion-based 3D object-detection methods, 3D datasets, sensors, 3D detection challenges, and possible research directions for autonomous driving. Recently, Alaba and Ball [24] also reviewed an image 3D object detection for autonomous driving. The 3D object-detection evaluation techniques, 3D bounding box encoding techniques, and image-based (RGB and stereo) object detection works for autonomous driving were reviewed.

This survey presents point cloud encoding techniques, 3D coordinate systems in 3D object detection, and others not covered in the previous survey papers, including recently published works (For 3D bounding box encoding techniques, 3D detection evaluation methods, sensors in autonomous driving, radar-related 3D object-detection methods, 3D object-detection challenges, possible research directions, and datasets refer [3,24]). Most of the existing 3D object-detection methods reviewed general 2D and 3D detection methods and/or LiDAR and camera-based detections. This survey focuses on a detailed analysis of LiDAR-only methods. Few works reviewed LiDAR-only methods. However, we have included recently published works in addition to unique contributions, such as 3D coordinate systems in 3D object detection and stages of autonomous driving.

## 3. Background

This section presents feature-extraction techniques for point clouds, 3D coordinate systems for 3D object detection, and levels of autonomous driving.

### 3.1. Feature-Extraction Methods

Feature extraction plays a crucial role in detection and classification tasks. Before the DL era, image features were usually extracted using handcrafted feature extractors based on aspects such as texture, color, and shape. On the other hand, DL networks can extract features from images without prior engineered data. Optimal feature learning is important to achieve optimal performance. The Harris interest point detector [25], Shi-Tomasi corner detector [26], Scale Invariant Feature Transform (SIFT) [27], and Speed-Up Robust Features (SURF) [28] methods are commonly used handcrafted feature extractors. In different computer vision applications [29,30,31], convolutional neural networks can replace these traditional feature extractors because of their ability to extract complex features and learn features efficiently. The image feature-extraction networks use different backbone networks such as AlexNet [32], VGGNet [33], GoogleNet [34], ResNet [35] Inception-ResNet-V2 [36], MobileNet [37], and DarkNet-19 [38]. The point cloud representations differ from images because of the unstructured nature and sparsity of points, which makes their feature-extraction networks different from image feature extractors. We can categorize feature extractors of LiDAR point clouds as point-wise, segment-wise, object-wise, and CNN-based networks [21] (see the details in Section 4).

Point-wise feature extractors take the whole point cloud as an input and then process, analyze, and label each point individually, such as PointNet [12] and PointNet++ [13]. Segment-wise feature extractors segment point clouds into volumetric representations and stack the volumetric representations to form LiDAR point clouds such as voxels [9,39,40], pillars [41], and frustums [12]. The point-wise and segment-wise feature extractors extract features without prior knowledge of whether the point or voxel belongs to an object. The object-wise feature extractors project LiDAR point clouds into 2D representations and generate the object proposals using 2D networks. Then, they regress the region proposals to predict the 3D bounding boxes. MV3D [7] uses the object-wise feature extractor to generate object proposals from LiDAR point cloud and fuse multiview features. On the other hand, CNN-based feature extractors use CNN to learn features from LiDAR data, such as images. These CNN-based feature extractors can be 2D backbone [32,33,34,35,36], 3D backbone sparse convolutional networks [42,43], CNN networks based on voting scheme [44], and graph convolution network [45].

The 3D backbone feature extractors are directly applied to 3D space using 3D convolutions. However, these networks consume large amounts of memory and take a long time to process the points. The LiDAR point cloud data are sparse and unstructured. Applying a standard convolution to these data points is inefficient because most areas are empty, and time is wasted on areas that do not have relevant information. The sparse convolution solves this problem by reducing the number of points to be processed and exploiting the areas with point cloud data only, which facilitates feature extraction and saves memory. Graham [42,43] developed sparse convolution by processing only areas with relevant information to save computational power. Each hidden value with zero input is considered a ground state, but the ground state is nonzero because of the biased terms. Then, when the inputs are sparse, the hidden variables that differ from their ground state are considered for calculation. Sub-manifold sparse convolutional networks (SS-CNs) [46] developed as an efficient sparse convolution for sparse 3D operation. The work considers feature vectors active if the ground state is nonzero, which is similar to [42,43].

Wang et al. [44] proposed a voting-based sliding window feature-extraction technique. First, the 3D point clouds are discretized into a grid at a fixed resolution. Then, the feature vectors are extracted from occupied cells. This method applies filters only on the occupied grid rather than the entire grid. The 3D detection window of fixed size is placed on the feature grid and passed into the classifier, such as SVM. Finally, the classifier decides the presence of an object by returning the detection score. Then, Vote3deep [47] further improves voting-based feature extractions by proposing a feature-based feature extraction and L1 penalty on filter activation intermediate feature representations.

Similarly, the graph convolution network (GCN) operates similarly to CNN but encodes points as nodes and nodes connected through edges. Applying GCN operation to a set of neighborhood nodes extracts features. EdgeConv [45] applies dynamically updated graph convolutions on the edges of local geometric structure points of *K*-nearest neighboring pairs. Then, they apply a multilayer perceptron to extract edge features from each point. DeepMRGCN [48] proposed a memory-efficient GCN model for feature extraction of non-Euclidean data.

### 3.2. Coordinate Systems

Due to the variety of sensors used in 3D detection, there are different coordinate systems. Therefore, different 3D datasets follow different data formats. Although there are a variety of datasets and sensors, coordinate systems in 3D object detection can be categorized into three [49].

**Figure 1:**
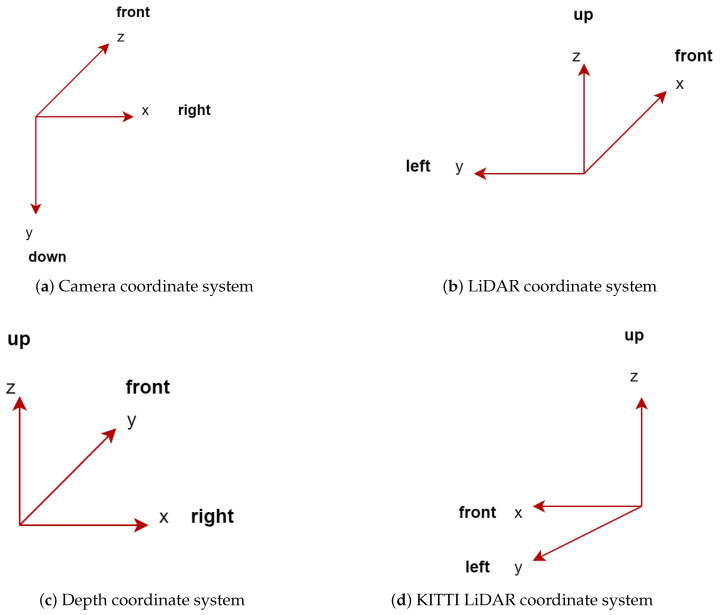
3D object-detection coordinate systems. The KITTI camera coordinate system is similar to the camera coordinate system (**a**). However, its LiDAR coordinate system *x*-coordinate differs from the commonly used LiDAR *x*- coordinate system (compare (**b**,**d**)).

1. **Camera coordinate system:** In this coordinate system, the positive direction of the *x*-axis points to the right, the positive direction of the *y*-axis points to the ground, and the positive direction of the *z*-axis points to the front. Figure 1a shows a camera coordinate system.

2. **LiDAR coordinate system:** In the LiDAR coordinate system, the positive direction of the *x*-axis points to the front, the positive direction of the *y*-axis points to the left, and the negative direction of the *z*-axis points to the ground as shown in Figure 1b.

3. **Depth coordinate system:** In the depth coordinate system, the positive direction of the *x*-axis points to the right, the positive direction of the *y*-axis points to the front and the negative direction of the *z*-axis points to the ground. Three-dimensional object-detection networks, such as VoteNet [50], H3DNet [51], etc., used the depth coordinate system. The depth coordinate system is shown in Figure 1c.

The commonly used dataset, for example, KITTI [52] camera and LiDAR coordinate, are shown in Figure 1a,d, respectively. In multisensor fusion-based or point cloud-based methods, the coordinate transformation is necessary from the beginning for data preprocessing, such as data augmentation. We do not cover the detailed mathematical derivation of coordinate transformation, but we give the intuitions and basic concepts of coordinate transformations between LiDAR and the camera. For example, for a homogeneous 3D point, *p* = ${\left(x,y,z,1\right)}^{T}$ in rectified (rotated) camera coordinates, the $i^{th}$ camera image corresponding point y = ${\left(u,v,1\right)}^{T}$ can be expressed as:

(1) $$y=p_{rect}^{\left(i\right)}p,$$

where *i* ϵ 0, 1, 2, 3 is the camera index (four cameras with centers aligned to the same *x/y*-plane in the KITTI dataset), and camera 0 is a reference camera.

$p_{rect}^{\left(i\right)}\epsilon R^{3\times 4}$ is $i^{th}$ the projection matrix after rectification, which can be given as:

(2) $$p_{rect}^{\left(i\right)}=(\begin{array}{cccc} f_{u}^{\left(i\right)} & 0 & c_{u}^{\left(i\right)} & -f_{u}^{\left(i\right)}b_{x}^{\left(i\right)}\\ 0 & f_{u}^{\left(i\right)} & c_{u}^{\left(i\right)} & 0\\ 0 & 0 & 1 & 0 \end{array}),$$

where $b_{x}^{\left(i\right)}$ is the baseline (in meters) with respect to a reference camera 0. To project the reference camera coordinates 3D points in *p* to the $i^{th}$ image plane in point y, the rectifying matrix of the reference camera *R* (0) should be considered. Thus:

(3) $$y=p_{rect}^{\left(i\right)}R_{rect}^{\left(0\right)}p,$$

For 3D point, *p* in LiDAR coordinates the corresponding point y in the $i^{th}$ camera image can be given as:

(4) $$y=p_{rect}^{\left(i\right)}R_{rect}^{\left(0\right)}T_{velo}^{cam}p,$$

$T_{velo}^{cam}\epsilon R^{3\times 4}$ is the rigid body transformation from Velodyne LiDAR to camera coordinates and $R_{rect}^{\left(0\right)}\epsilon R^{3\times 3}$ is the rectifying rotation matrix (Ref [52,53] for more details).

### 3.3. Stages of Autonomous Driving

Automated vehicles should have a safety-critical control function, such as steering, throttle, or braking, that can occur without direct driver intervention [54]. Vehicles that give safety warnings to drivers, such as forward crash warnings, are not considered automated unless they perform control functions even though the necessary data are received, processed, and the warning is given without driver intervention. When we think of autonomous driving, most people think of fully autonomous driving; however, autonomous driving has different stages. The vehicle automation levels range from level zero (no automation) to level five (fully autonomous). We present levels of autonomous driving for general understanding and to give insight into what should be needed for achieving fully autonomous driving. Vehicles should be equipped with multiple sensors to achieve robust driving, especially for levels four and five. However, this survey presents only LiDAR-related works (Refer [3] for different sensors used in autonomous driving). Therefore, the main goal of autonomous driving should be achieving level four and level five driving.

**Level zero–No Automation:** The driver is in complete control of driving and is responsible for monitoring the roadway. The vehicles with some driver support, such as steering, braking, etc., do not take action or do not have control authority over the vehicle. Therefore, it is considered no automation. Forward collision warning, lane departure warning, and blind spot monitoring driver support systems are considered level zero automation.

**Level one–Function-specific Automation/Driver Assistance:** In this level of automation, the driver has complete control and is solely responsible for the vehicle’s safe operation. The system can perform a single automated system, such as steering or acceleration (as in adaptive cruise control), but not both. Drivers can take off their feet from the pedals so that it is sometimes called feet off level. Each system operates independently if a vehicle is equipped with multiple automated systems, such as lane keeping, steering, and acceleration. The automated system only supports the driver and does not replace the driver.

**Level two–Partial Driving Automation:** The driver is responsible for controlling the vehicle and monitoring the roadway. However, the system can perform two control functions: steering and acceleration. In this level of automation, the driver can be free from operating the steering wheel and pedal simultaneously. The driver engages whenever necessary to control both or one of the control functions. The driver can take off both feet and hands, called hand off level.

**Level three–Conditional Automation:** At this level of automation, vehicles can perform most of the tasks under specific traffic or environmental conditions (usually in urban areas with low-speed driving, such as 25 mph). The driver can override the system anytime. Therefore, the driver remains ready to take control when the system occasionally signals the driver to reengage in the driving task. The driver has sufficient transition time to control the vehicle. The driver can disengage from driving. Therefore, it is called eyes off level.

**Level four–High Automation:** At this level of automation, the vehicle can perform all the control functions under specific conditions. The driver does not engage in driving the vehicle but can override it. It is called mind off level. These technologies are not commercially available yet.

**Figure 2:**
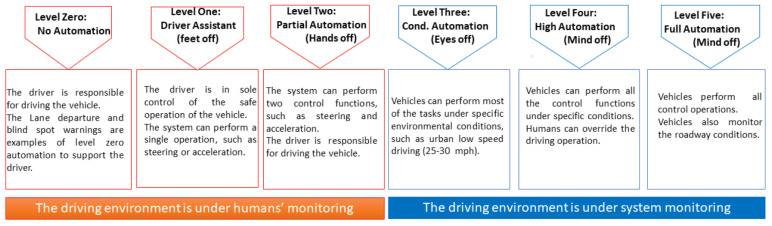
The levels of autonomous driving. In the first three levels (level zero to level two), the humans monitor the driving environment, whereas the system monitors the driving environment for the last three levels of driving.

**Level five–Full Automation:** The vehicle performs all control operations and monitors roadway conditions without human intervention. The driver may provide navigation input, but all the controlling operations, including safe operation and roadway safety, rest on the vehicle. This level is also called mind off because vehicles can be without humans, or humans can do other activities. The diagrammatic representation of the levels of autonomous driving is shown in Figure 2.

The full automation level of autonomy replaces a human driver. The following four areas are vital to replacing human drivers in autonomous driving [55]:

1. **Vehicle Location and Environment:** For fully autonomous driving without human intervention, precise and accurate information about the driving environment must know the road signs, pedestrians, traffic, and others.
2. **Prediction and Decision Algorithms:** An efficient deep or machine learning algorithm is needed to detect, predict, and decide when interacting with other vehicles, pedestrians, and situations.
3. **High Accuracy and Real-time Maps:** Detailed, precise, and complete maps are needed to obtain information about the driving environment for path and trajectory planning.
4. **Vehicle Driver Interface:** Smooth and self-adaptive transition to/from the driver and an effective way to keep the driver alert and ready is needed, which increases customer satisfaction and confidence, especially at the beginning of the technology.

To replace human drivers, we need efficient sensors that can do a human eye’s task, maps to do the human memory, ML algorithms to make the human brain decision, and vehicles to *x* communication such as human ears.

## 4. LiDAR 3D Object-Detection Methods

Although cameras are inexpensive, they lack the ability to create precise depth information for accurate 3D object detection. Additionally, cameras are vulnerable to adverse weather, such as snow, fog, and rain [3,24]. Point cloud-based methods provide solutions for such problems to improve performance significantly because sensors, such as LiDARs and radars, provide depth information, which is essential for accurate object size and precise location estimation. However, point cloud data are sparse, unordered, unstructured, and unevenly distributed. Therefore, they cannot fit directly into image-based deep-learning methods and need a different processing method for image-processing techniques. Different techniques have been proposed to reduce the sparsity of point cloud data, including dilated convolution [56] and flex convolution [57]. Yu et al. [56] put forth dilated convolution for dense prediction and enlarging receptive field. Although dilated convolution enlarges the receptive field and improves performance, it suffers from a gridding effect [58], which causes information discontinuity because of combination pixels from different neighbors [59]. Groh et al. [57] also developed flex-convolution to process irregular data, such as point clouds. We divide the point cloud representation methods into projection, voxel, and raw point cloud.

### 4.1. Projection Methods

The projection method transforms the 3D data points into a 2D space using plane (image) [60], spherical [61], cylindrical [62], or bird’s-eye view (BEV) [7] projection techniques. The projected data can be processed using the standard 2D methods and regressed to obtain the 3D bounding boxes. It is hard to detect occluded objects in 2D plane representations. Additionally, the 2D plane representations cannot keep object length and width. Tian et al. [6] presented a range image-based 3D detection model. The network uses the projected range image to generate multilevel FPN features and feed the output to the detection head for classification and regression of 3D boxes. The model was trained and tested on the nuScenes [63] dataset. When a point cloud is projected to a range image, occlusion and scale change may reduce the detection performance, which is not the case when projected to BEV representation. Therefore, solving the occlusion and scale change problems would improve the model as with other representations, such as BEV.

The BEV representation, mostly encoded by height, intensity (reflectance value), and density, solves these problems because, in BEV (top view) representation, objects occupy separate spaces on the map. Therefore, it avoids occlusion and scale problems [7]. The BEV lies on the ground plane so that the variance in the vertical direction is small, which helps to obtain accurate 3D bounding boxes compared to other projection methods. Because of these reasons, BEV is mostly used as a LiDAR 2D representation for 3D detection. However, the BEV representation causes height compression at each position, which may cause semantic ambiguity.

**Figure 3:**
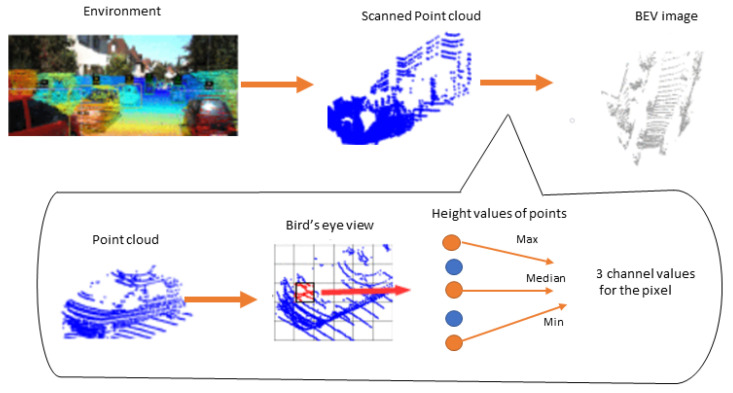
BEV image construction [64].

Yu et al. [64] transformed point cloud data into BEV elevation images by encoding each pixel using maximal, median, and minimal height values as shown in Figure 3.

**Figure 4:**
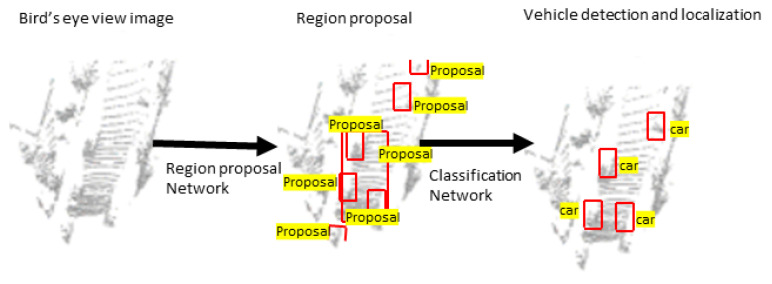
Two-stage vehicle detector [64].

The BEV pixel of the image represents the ground coordinates so that it simultaneously detects and localizes vehicles in the ground coordinates. The classification of vehicle and localization is shown in Figure 4. Wirges et al. [65] encoded BEV using intensity, height, detections, observation, and decay rate. Similarly, BirdNet [66] and BirdNet+ [67] encoded BEV using height, intensity, and density information. The proposed density normalization map normalizes the number of points collected by different LiDAR sensors. BirdNet+ adds an improvised regression method to avoid the post-processing task in BirdNet to obtain the 3D boxes. Barrera et al. [68] modified BirdNet+ [67] and proposed a two-stage model using a Faster R-CNN [69] model. The LIDAR point cloud data are projected into BEV and normalized using the proposed density encoding method. The ResNet-50 [35] with FPN [70] is used as a backbone network and RPN as candidate proposals generation network. An ad-hoc set of nine anchors are employed to avoid the size constraints of objects in BEV. The category classification, 2D BEV rotated box regression, and 3D box regression is performed by the detection head’s next stage. Even though the model shows a good performance on the KITTI [52] and nuScences [63] datasets, it is far from the real-time implementation due to slow speed detection. V and Pankaj proposed YOLO3D [71] model, an extension of YOLOv4 [72] 2D model. The point cloud data are projected onto the BEV domain before feeding to YOLOv4. Then, Euler-Region-Proposal Network is used to predict the 3D bounding box information.

Chen et al. [73] put forth a real-time multiclass 3D scene understanding two-stage model called MVLidarNet using multiple views. In the first stage, the point cloud data are projected into a perspective view to extract semantic information. In the second stage, the processed point cloud data are projected into BEV representation, which is important for classifying and detecting objects. An FPN-like encoder-decoder architecture is used in both stages. To detect individual object instances, DBSCAN [74] clustering algorithm is used. The model trained on the SemanticKITTI [75] dataset for semantic segmentation and KITTI [52] dataset for detection. The model detects objects and simultaneously determines the derivable space. Lu et al. [76] proposed the Range-Aware Attention Network (RAANet), which extracts more powerful BEV features and improves a 3D detection. Even though far-away objects have sparser LiDAR points, they do not appear smaller in the BEV representation. This weakens BEV feature extraction using shared-weight convolutional neural networks. The authors proposed RAANET to solve this challenge and extract more powerful features to enhance the overall 3D detection. They proposed the RAAConv layer instead of the Conv2D layer to extract more representative BEV features. They also developed an auxiliary loss of density estimation to enhance the detection of occlusion-related features. Even though the authors claimed the model is lightweight, the lite version runs a maximum of 22 Hz, which is far from the real-time implementation. We expect more lightweight models that can run at a higher frequency (Hz) for real-time implementation.

Du et al. [77] developed a detection network to provide an accurate 3D detection result. A two-stage CNN is presented for the final 3D box regression and classification based on the inputs fit into the 3D bounding box. PIXOR [78] is a proposal-free single-stage detector that balances high accuracy and real-time efficiency using a BEV representation of the point cloud. The network outputs a pixel-wise prediction of region proposals. Yang et al. put forth PIXOR++ [79], a single-stage 3D object-detection model. The model extracts geometric and semantic features from HD maps. A map prediction module is also proposed to estimate the map from raw LiDAR data. The model was trained and tested on the KITTI [52] dataset. Wenjie et al. [80] also proposed a deep NN model that jointly learns 3D detection, tracking, and motion forecasting by exploiting the BEV representation. The model is robust to occlusion and sparse data. Similarly, Complex-YOLO [81] extended the YOLOv2 [38] 2D image-based detection model for 3D object detection using a complex angle regression strategy for multiclass 3D box estimation. The introduced specific Euler-Region-Proposal Network (E-RPN) estimates the orientation of objects accurately by adding an imaginary and a real fraction for each bounding box. The model runs over 50 frames per second (fps), which is convenient for real-time operation. YOLO3D [82] also extended the loss function of YOLOv2 [38] using the yaw angle, 3D object bounding box center coordinates, and height of the box as a direct regression method.

### 4.2. Volumetric (Voxel) Methods

The volumetric method discretizes the unstructured and sparse 3D point clouds into a volumetric 3D grid of voxels (volume elements) and uses a series of voxels to represent a 3D point cloud. Voxels are similar to image pixels, but in 3D representation that explicitly provides depth information. Because of the 3D point clouds’ sparsity nature, most volumes are empty. Consequently, processing the empty cells reduces performance. Another limitation of the voxel representation is that it uses 3D convolution for CNN models, increasing the computational cost and involving a trade-off between resolution and memory. In this section, we reviewed voxel- and pillar-based methods. Wang et al. [44] proposed a voting scheme-based sliding window approach, vote3D, for 3D detection to minimize the effect of the sparsity of point clouds.

Similarly, Sedaghat et al. [83] introduced a category-level classification network that estimates both the orientation and the class label. The orientation estimation during training improves the classification results during test time. Vote3Deep [47] presented the first sparse convolutional layers based on a feature-centric voting scheme [44] to leverage the sparsity point cloud. The computational cost is proportional to the number of occupied cells rather than the total number of cells in a 3D grid.

**Figure 5:**
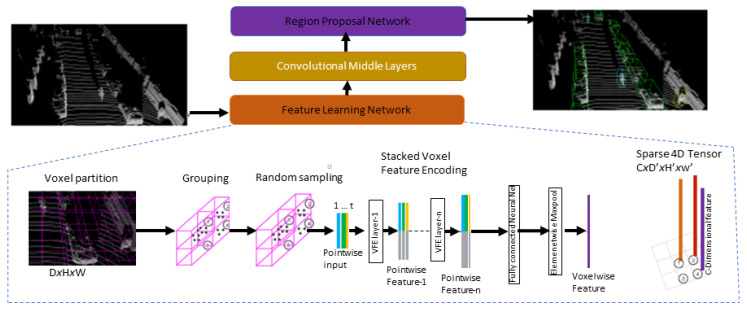
VoxelNet Architecture [9]. The raw point cloud is partitioned into voxels and transformed into vector representation by the feature learning network. The convolutional middle layers process the 4D tensor vector before the region proposal network generates 3D detection.

Likewise, Li put forth a 3DFCN [84] model for 3D object detection. They extended the 2DFCN [85] into a 3D operation on voxelized 3D data. The point clouds are discretized on a square grid with a 4D array representation using dimensions of length, width, height, and channels. Zhou and Tuzel created a VoxelNet [9] network for 3D object detection. The introduced voxel feature-encoding (VFE) layer learns a unified representation for each group of points within voxels instead of using handcrafted features as shown in Figure 5. Then, the region proposal network (RPN) takes the output of the volumetric representation and VFE layer output convolved with 3D convolutions to generate detections. Converting point clouds into a dense tensor structure help to implement stacked VFE operations in parallel across points efficiently. Processing only the nonempty voxels rather than the whole voxels in a grid improves the performance.

Furthermore, SECOND [39], a spatially sparse convolution network to extract information from LiDAR data, is introduced. The sparse convolution network uses a GPU-based rule-generation algorithm to increase the speed of operation. The work outperforms the previous results, such as VoxelNet. HVNet [86] is a hybrid single-stage LiDAR-based 3D detection network. The network fuses a multiscale VFE at a point-wise level using FPN [70]. Then, the result is projected into multiple pseudo-image feature maps using attentive VFE (AVFE) to solve the performance issues because of the size of the voxels. A small voxel improves performance, but the detection speed is slow. Large voxel sizes cannot capture the features of small objects. It uses a multiscale fusion network to solve these problems. The performance result shows a state-of-the-art mAP performance on the KITTI dataset.

Du et al. put forth Associate-3Ddet [87] model to learn the association between perceptual features extracted from real scenes using a perceptual voxel feature extractor (PFE) and conceptual features generated from augmented scenes using a conceptual feature generator (CFG) based on domain adaptation. First, CFG is separately trained on the conceptual scene and integrated with complete object models. The perceptual-to-conceptual module (P2C) uses an incompletion-aware re-weighting map to build associations between perceptual and conceptual features. Once the perceptual and conceptual domains are well aligned after training, the network can adaptively generate the conceptual features without CFG. The models show promising results on the benchmarks of the KITTI [52] dataset. Liu et al. proposed TANet [88], a robust network comprised of Stacked Triple Attention (STA) and Coarse-to-Fine Regression (CFA) modules. The STA module obtains multilevel feature attention by performing a stack operation on channel-wise, point-wise, and voxel-wise attention. The CFA module helps to achieve more accurate detection boxes without more outrageous computational costs. Then, the pyramid sampling aggregation (PSA) module takes the output of CFA and provides cross-layer feature maps. The cross-layer feature maps capture multilevel information, which has larger receptive fields with richer semantic information from the high-level features and larger resolution from low-level features. This helps to obtain more robust representative features for objects and enhances the detection capability.

Deng et al. put forth Voxel R-CNN [89], a voxel-based 3D detection model comprising a 3D backbone network, a 2D BEV RPN, and a detection head. The point cloud data are voxelized and fed into the 3D backbone network for feature extraction. Then, the features are converted into BEV representation before applying the 2D backbone and RPN for region proposal generation. ROI features are extracted from the 3D features by applying voxel RoI pooling. Finally, the detection head uses RoI features for box refinement. The experiments are conducted on the KITTI [52] and Waymo Open [90] datasets. Li et al. proposed SIENet [91], a two-stage spatial information enhancement network for 3D object detection. The authors designed a hybrid-paradigm region proposal network (HP-RPN), which includes the SPConv branch, the auxiliary branch, and the key-point branch, to learn discriminative features and generate accurate proposals for the spatial information enhancement (SIE) module. The SPConv branch learns more abstract voxel features from the voxelized point cloud. Concurrently, the corresponding voxel features are dynamically encoded by the key-point branch. The auxiliary branch is used to learn object structures. The spatial shapes of the foreground points in the candidate boxes are predicted using the proposed SIE module. The SIE module learns structure information to enhance the features for box refinement. The model was trained and tested on the KITTI [52] dataset.

Liu et al. [92] put forth a single-stage sparse multiscale voxel feature aggregation network (SMS-Net). The model comprises sparse multiscale-fusion (SMSF) and shallow-to-deep regression (SDR) modules. The SMSF module fuses point-wise and multiscale features at the 3D sparse feature-map level to achieve more fine-grained shape information. The SDR improves the localization and 3D box estimation accuracy through multiple aggregations at the feature-map level with less computational overhead. The model shows the comparable result on the KITTI [52] dataset. Sun et al. [93] proposed a semantic-aware 3D object-detection model. The proposed voxel-wise class-aware segmentation module learns the fine-grained semantic features. Additionally, the semantic-aware refinement module generates coarse proposals. The model shows a competitive result on the KITTI [52] dataset, especially for small objects.

Liu et al. proposed MA-MFFC [94], attention and multiscale feature fusion network with ConvNeXt module for 3D detection network. The multi-attention module comprises point-channel and voxel attention to enhance key point information in voxels and obtain more robust and discriminative voxel features in the 3D backbone. The convolutional layer is replaced with a ConvNeXt module to extract richer features more accurately. The experimental result on the KITTI [52] dataset shows an improvement over Voxel R-CNN [89] baseline network. Wang et al. put forth a self-attention graph convolutional network (SAT-GCN) [95] for 3D object detection. The proposed model consists of three modules. The first vertex feature-extraction (VFE) module with GCN decodes point cloud data and extracts local relationships between features. The second module, self-attention with dimension reduction, uses self-attention to further enhance the neighboring relationship between features. The final module, far-distance feature suppression, generates global features by suppressing far-away features. The experimental result on the KITTI [52] and nuScenes [63] datasets show the effectiveness of the proposed model. Fan et al. [96] proposed voxel-based fully sparse 3D object detector. The sparse instance recognition module generates instance features by grouping points before the prediction is employed. Grouping points into instances reduces the missing center feature, such as center-based detection and neighbor queries. The model shows competitive performance on the Waymo [90] dataset.

Li et al. [91] presented a two-stage model with a spatial information enhancement network. The first stage of the hybrid-paradigm RPN consists of the SPConv branch, the auxiliary branch, and the key-point branch to extract features and generate proposals. The second stage predicts the 3D bounding boxes via the spatial information enhancement module. The number of LiDAR points of an object decreases with distance. The spatial information enhancement module helps predict the spatial shapes of objects. It extracts the structure information and the representative features for further box refinement, which increases the detection accuracy of far-away objects. Hu et al. [97] proposed a two-stage point density-aware voxel (PDV) network to account the point density variations. The PDV with voxel point centroids localizes voxel features from the 3D sparse convolution backbone. The density-aware ROI-grid pooling module using kernel density estimation (KDE) and self-attention with point density positional encoding aggregates the localized features. The model was trained and tested on the Waymo [90] and KITTI [52] datasets.

Another work, Frustum PointNets (F-PointNet) [10], is a cascaded fusion network for 3D detection and semantic segmentation. The 2D region proposals are generated from RGB images using CNN and extruded to 3D region proposals called Frustum point clouds. Then, the Frustum PointNet performs 3D object instance segmentation and amodal 3D bounding box regression. Although the network shows incredible performance, there are failures because of inaccurate pose and size estimation in the sparse point cloud. The network also has a limitation when there are multiple instances from the same category and dark lighting or strong occlusion. Cao et al. [11] proposed multiview Frustum PointNet (MVFP), which is an extension of the F-PointNet [10] to reduce the rate of miss detection. F-PointNet [10] misses detection when the RGB feature detector does not capture all the information. MVFP added an auxiliary BEV detection to handle the miss detection. The authors use an IOU [98] to match the F-PointNet [10] 3D and BEV 2D bounding box. If there is a matching for the BEV box in the F-PointNet box, the object is detected successfully by F-PointNet [10]. Therefore, the F-PointNet result is the output. On the other hand, if there is no match for the BEV box, the object is possibly miss-detected. Thus, the 2D box in BEV will be fed back to the raw point cloud. The experimental result on the KITTI [52] dataset shows the model outperforms the F-PointNet [10].

Wang and Jia put forth Frustum-ConvNet [99], an amodal 3D model by sliding frustums to aggregate local point-wise features. The authors proposed a method to obtain a sequence of frustums by sliding a pair of planes along the Frustum axis. The pair of planes are perpendicular to the Frustum axis as well as the optical axis of the camera is perpendicular to the 2D region proposal. For each 2D region proposal, a sequence of frustums is generated, and all points inside the Frustum are grouped. Point-wise features inside each Frustum are aggregated as a Frustum-level feature vector. Then, Frustum-level feature extraction is undertaken using PointNet [12]. The Frustum-level feature vectors array as a 2D feature map and feed into a fully convolutional network before feeding to the detection head, which estimates oriented 3D bounding boxes. Finally, the proposed refinement network applies to ensure the predicted box precisely bounds object instances. The network was trained on the KITTI [52] and indoor SUN-RGBD [100] datasets.

Some works discretize point clouds into vertical columns called pillars instead of voxels. Unlike voxels, pillars are suitable for 2D convolutions. Pointpillars [41] is a single-stage 3D object-detection method by learning the features on pillars (vertical columns) to predict 3D oriented boxes for objects. It is a point cloud encoding technique by learning the features rather than using fixed encoders. It can run at 105 Hz and outperform previous detectors, such as SECOND [39], in BEV and 3D KITTI detection benchmarks. McCrae and Zakhor [101] modified PointPillars [41] as a recurrent network using fewer LiDAR frames per forward pass. The ConvLSTM layer is inserted between PointPillars [41] backbone and detection head to propagate information through time. The network takes fewer LiDAR frames than PointPillars [41], which reduces complexity by processing fewer data. The model outperforms the PointPillars [41] network, which uses 10 LiDAR frames per forwarding pass, with only three LiDAR frames per forwarding pass. However, there was a performance decline in the vehicle class. Detecting small objects is one of the main challenges in autonomous driving, and decreasing the LiDAR frames will further hurt performance.

Wang et al. [102] proposed an anchor-free pillar-based model for autonomous driving. As in other methods, the network predicts bounding box parameters per pillar. The authors also include an interpolation method in pillar-to-point projection to improve the final prediction. The model is evaluated on the Waymo [90] dataset. Fan et al. [103] proposed a single-stride sparse transformer network to avoid information loss because of multiple strides using PointPillars as a base network. The model was trained and tested on the Waymo [90] dataset. Tong et al. put forth ASCNet [104], a two-stage network. Pillar-wise spatial-context feature-encoding and length-adaptive RNN-based modules are proposed to learn features from point clouds and to solve the inhomogeneity in point clouds, such as a varying number of points in the pillars, the diverse size of Regions of Interest (RoI), respectively. The model shows competitive performance on the KITTI [52] dataset.

Zhang et al. [105] put forth a semisupervised pillar-based single-stage model by adopting a teacher-student framework. The authors used the same loss function as SECOND [39] and PointPillars [41]. The exponential moving average (EMA) teacher and asymmetric data augmentation method are used to improve the efficiency of a teacher-student network. Even though the performance of this model is not good enough for real-time application, it is a suitable model to start with by developing a semisupervised model to reduce the time and cost of dataset labeling and annotation. Caine et al. [106] put forth a pseudo-labeling domain adaptation method using PointPillars [41] as a student network. The teacher network pseudo-labels all unseen source data, and the student network trains with a union of labeled and pseudo-labeled data. Finally, the student and teacher networks were evaluated on the Waymo Open Dataset [90] and Kirkland validation split [107].

Bai et al. proposed PillarGrid [108], a cooperative perception model that fuses information from multiple 3D on-board and roadside 3D LiDARs. The PillarGrid model consists of four main components: (1) cooperative preprocessing for point cloud data transformation, (2) pillar-wise voxelization and feature extraction, (3) grid-wise deep fusion to fuse deep features, and (4) CNN-based augmented 3D object detection. The model was trained and tested on a dataset collected using CARLA [109]. Lin et al. [110] put forth a pointpillar-based 3D object-detection module to improve the performance for snow weather conditions. The proposed double-attention module is used to reweight the input features of pillars feature extraction. The feature refinement extraction module captures context information to reduce the noise of local features. Finally, the proposed maximum mean discrepancy module is employed to obtain the domain feature representation distribution. The module is trained and tested on the Canadian Adverse Driving Condition [111] and KITTI [52] datasets. Recently, Alaba and Ball proposed WCNN3D [59], a wavelet-based 3D object-detection network. The model comprises discrete wavelet transform (DWT) and inverse wavelet transform (IWT) with skip connection between the contrasting layers, expanding layers, and the previous layers in the model. The model is designed without the pooling operation to reduce the information loss during downsampling. The DWT is used as a downsampling operator, whereas IWT is an upsampling operator. The wavelet’s lossless property helped recover the lost details during the downsampling operation. The experimental result on the KITTI [52] dataset shows the model outperforms pillar-based models, such as PointPillars [41], and PV-RCNN [112], and is more suitable for the detection of small objects such as pedestrians and cyclists.

### 4.3. Raw Point Cloud Methods

**Figure 6:**
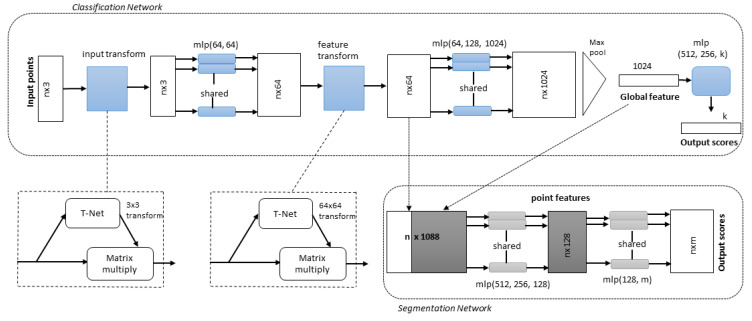
PointNet Architecture [12]. The classification network transforms n inputs into aggregate point features using feature transformation. It outputs *k* class classification scores. Then, the segmentation network concatenates the local and global features and generates per-point scores. The multilayer perceptrons (mlp) with layer size in brackets are also given.

The LiDAR data projection and volumetric methods cause spatial information loss during conversion to another domain, so processing point clouds directly are important to keep this spatial information. However, the raw point cloud methods have high sparsity and computational costs due to 3D convolutions. PointNet [12] is a unified architecture for 3D object classification, part segmentation, and semantic segmentation that directly uses raw point cloud data, as shown in Figure 6. The classification network transforms n inputs into aggregate point features using feature transformation. It outputs *k* class classification scores. Then, the segmentation network concatenates the local and global features and generates per-point scores.

The network applies feature transformation to each input and aggregates point features. The process generates classification scores for each class. Then, global features, local features, and outputs-per-point scores are aggregated for segmentation. Although the PointNet model is promising, it does not capture local structures, which is crucial for fine-grain patterns and better generalizability for unseen cases.

Qi et al. [13] developed PointNet++ to solve the PointNet architecture limitations by processing a set of points hierarchically sampled in a metric space. Distance metrics are used to partition the set of points into overlapping local regions by leveraging neighborhoods at multiple scales and centroid locations using the farthest point sampling (FPS) algorithm and then learning the local features using PointNet. Finally, multiscale grouping forms multiscale features by concatenating features at different scales. Multiresolution grouping adaptively aggregates information based on the distributional properties of points. It minimizes computational costs more than multiscale grouping.

Likewise, Shi et al. presented PointRCNN [14], a two-stage model using raw point cloud data. The first stage generates 3D proposals by segmenting the point clouds into foreground points and background bottom-up. The second stage refines the 3D bounding box proposals in the canonical coordinates to achieve better detection results. In the second stage, the authors adopted a pooling operation to pool learned point representations from proposal generation and transformed them into canonical coordinates. The canonical coordinates are combined with the pooled point features and the segmentation mask from the first stage to learn relative coordinate refinement and use all information from the first stage segmentation and proposal sub-network. They also proposed bin-based loss for efficient and effective 3D bounding box regression. The model was trained and tested on the KITTI [52] dataset.

**Figure 7:**
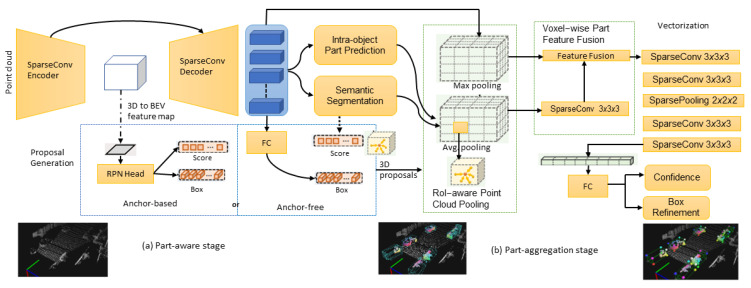
The part-aware and aggregation proposed 3D object-detection network architecture [113]. The model consists of two parts: (**a**) The intra-object part locations are predicted by the part-aware network. Then, 3D proposals are generated before feeding to the encoder-decoder network. (**b**) ROI-aware pooling is undertaken in the part-aggregation stage.

Shi et al. [113] extended the pointRCNN [14] model by proposing a part-aware and aggregation neural network (Part-A2) as shown in Figure 7. The part-aware network learns to estimate the intra-object part locations of foreground points and generate 3D proposals simultaneously by extracting discriminative features from the point cloud. Intra-object part locations are the relative locations of the 3D foreground points regarding their corresponding ground-truth boxes.

The discriminative point-wise features for foreground point segmentation and intra-object part location estimation are extracted using an encoder-decoder network with sparse convolution and deconvolution [46,114]. The model acquires the potential to infer the shape and pose of objects by learning to estimate the foreground segmentation mask and the intra-object part location of each point. The authors used anchor-free and anchor-based methods for 3D proposal generation. The anchor-free method is more memory efficient, whereas the anchor-based method gives better recall with a more GPU memory cost. Once the intra-object part locations and 3D proposals are generated, box scoring and proposal refinement is completed by aggregating the part information and learning point-wise features of all the points within the same proposal. The canonical transformation reduces effects due to the rotation and location variations of 3D proposals. Then, the RoI-aware point cloud feature pooling module removes the ambiguity of the previous point cloud pooling operation. The box proposal scoring and refinement information is fused for fine detection results. The model outperforms PointRCNN on the KITTI [52] dataset.

Yang et al. [115] put forth a sparse-to-dense (STD) two-stage 3D object-detection framework. In the first stage, the bottom-up proposal generation network uses a raw point cloud as input and seeds each point with a new spherical anchor to generate proposals. Then a PointNet++ [13] backbone extracts semantic context features for each point and generates objectness scores to filter anchors. The authors proposed a PointsPool layer to generate features for each proposal and transform sparse, unstructured, and unordered point-wise proposals into more compact features. In the second stage, a prediction is made. To reduce inappropriate removal during post-processing, they introduced augmenting a 3D IOU branch for predicting 3D IOU between predictions and ground-truth bounding boxes. The result on the KITTI dataset [52] outperforms models, such as PointPillars [41] and PointRCNN [14].

Yu et al. [116] proposed an equivariant network with a rotation equivariance suspension design to achieve object-level equivariance for 3D detection. This method helps the bounding box independent of object pose and scene motion. The proposed method tested on different models, such as VoteNet [117] on the ScanNetV2 [118] dataset and transfer-based network [119] on the SUN RGB-D [100] dataset for indoor scenes and PointRCNN [14] on the KITTI [52] dataset for outdoor scenes. Furthermore, 3DSSD [120] is a lightweight and efficient 3D single-stage framework using point clouds. The feature propagation upsampling layers and refinement module, mostly common for point cloud-based models, were removed to reduce the computational cost. A fusion set of abstraction downsampling layers were proposed to keep important information for regression and classification tasks. Finally, a box prediction network and anchor-free regression head with a 3D center label were introduced to enhance the final performance. He et al. [121] developed a structure-aware single-stage 3D detection (SASSD) network. A detachable auxiliary network with point-level supervision was designed for better localization performance through learning the structure information and an efficient feature-map part-sensitive wrapping operation, PSWarp, to correct the misalignment between the predicted bounding boxes and corresponding confidence maps. This design solves the spatial loss due to downsampling in a fully convolutional operation. Gustafsson et al. [122] proposed conditional energy-based models by extending SASSD [121] model. The authors designed a differentiable pooling operator to regress 3D bounding boxes accurately. They integrated the differentiable pooling operation into the SASSD model, and the experimental result on the KITTI dataset [52] shows the model outperforms SASSD [121] model.

Similarly, Zheng et al. [123] proposed a confident IOU-aware single-stage object detector (CIA-SSD) network to solve the localization accuracy and classification confidence misalignment. The authors proposed a spatial-semantic feature aggregation module to adaptively fuse high-level abstract semantic and low-level spatial features. This fusion helps to predict bounding boxes and classification confidence accurately. An IOU-aware confidence rectification module further rectifies the predicted confidence for more consistent confidence with localization accuracy. The IOU-aware confidence rectification module solves the complexity of SASSD [121] because of the interpolation operation. A distance-variant IOU-weighted NMS module uses rectified confidence to obtain smoother regressions and avoid redundant predictions. The network shows a comparable performance for 3D car detection of the KITTI [52] dataset. Shi and Rajkumar proposed Point-GNN [124], a graph neural network-based model. A graph was constructed using vertices and connecting neighboring points within a fixed radius. Voxel downsampling was used to reduce the point cloud density, but the representation is still a graph. After the graph is constructed, a graph neural network is developed to refine the vertex features by aggregating features along the edges. An autoregistration mechanism was proposed to align neighbor coordinates and reduce translation variance. A box merging and scoring operation were also proposed to combine detection from multiple vertices with confidence scores. The model outperforms others, such as PointRCNN [14] and STD [115].

Zhou et al. [125] put forth a two-stage joint 3D semantic segmentation and detection model for autonomous driving. The model consists of two parts: spatial embedding (SE) learning-based object proposal and the refinement of local bounding boxes (BBoxes). Point-wise features (local features and global context information) are extracted using PointNet++ [13] as a backbone network, along with sampling and grouping. A SE method was proposed to assemble all foreground points into the corresponding object centers. Based on the SE results, the object proposals, instance segmentation, and BBox can be generated using a simple clustering strategy (*K*-means) [126]. Non-maximal suppression (NMS) is not employed in this model because only one proposal is generated for each cluster. Finally, the proposed instance-aware ROI pooling outputs refined 3D BBoxes and instance masks. The model was trained on the KIITI [52] dataset.

Mao et al. [127] put forth a two-stage model, pyramid R-CNN, which is compatible with voxels, points, and other representations of the LiDAR region of interest (ROI). A pyramid ROI head was proposed, which comprises an ROI-grid pyramid, ROI-grid attention, and Density-Aware Radius Prediction (DARP), to learn the features adaptively from the sparse points of interest. An ROI-grid pyramid collects points of interest for each ROI in the pyramid, which helps to mitigate the sparsity problem. The ROI-grid attention component incorporates conventional attention-based and graph-based points into a unified form to encode richer information from sparse points. Finally, the DARP module is dynamically adjusting the focusing range of ROIs to adapt to different point density levels. This model is robust to the sparse data and imbalanced classes. Yang et al. presented ST3D [128], a self-training 3D domain adaptive network. A 3D object augmentation technique, random object scaling (ROS), was developed to overcome the bias in object size in the labeled source domain. A quality-aware triplet memory bank (QTMB) was also proposed for pseudo-label generation and assessing pseudo-boxes’ quality. Finally, a curriculum data augmentation (CDA) strategy was developed using pseudo-labels by escalating the intensity of augmentation and simulating hard examples during training to overcome overfitting. This network trained with four datasets, namely KITTI [52], Waymo [90], nuScenes [63], and Lyft [129].

Hegde and Patel [130] proposed a source-free unsupervised domain adaptive model, which uses class prototypes to mitigate the effect of pseudo-label noise. The model performance dropped when tested with a dataset different from what was trained. This model minimizes the effect of domain change. During self-training, a transformer module was used to identify incorrect and overconfident annotation outliers and compute an attentive class prototype. Zheng et al. presented SE-SSD [131], a self-assembling single-stage 3D object detector. The CIA-SSD [123] model structure was used by removing the confidence function and DI-NMS. The SE-SSD framework consists of teacher SSD and student SSD. The teacher SSD receives the point of cloud input and produces a bounding box with confidence predictions. Then, the teacher supervises the student using the soft targets, which are the predictions after global transformations, with consistency loss to align the student predictions with soft targets. The teacher also supervises the student with hard targets using orientation-aware distance-IOU loss, focusing more on the alignment of box centers and the distance between the 3D centers of the predicted and ground-truth bounding boxes. The student parameters were updated using consistency loss and orientation-aware distance-IOU loss. In contrast, the teacher parameters were updated based on the student parameters with the standard exponential moving average (EMA) method. The model shows performance improvement on the KITTI [52] dataset over other methods, such as CIA-SSD [123].

Wang et al. [132] proposed a semisupervised network via temporal graph neural network. The teacher network takes a single point cloud frame input to generate candidate detections, which are input to the graph neural network to generate further refined detection scores. The generated pseudo-labels are then combined with the labeled point clouds to train the student’s model. The teacher network is updated in each step using the exponential moving average method. This semisupervised method is important to leverage abundant unlabeled data. The model was trained and tested on the datasets of the nuScenes [63] and H3D [133] datasets. Zhang et al. proposed PointDistiller [134] knowledge distillation method for point cloud data. The model includes the local distillation to extract and distills the local geometric structure of point clouds to the student network using dynamic graph convolution with a reweighted learning strategy to handle the sparsity and noise in point clouds. Transferring the teacher knowledge to the students on the point cloud data trained and tested both on voxel and raw point cloud detectors, such as PointPillars [41], SECOND [39], and PointRCNN [14].

Wang et al. put forth POAT-Net [135], a parallel offset-attention assisted transformer model. The parallel offset-attention method helps to capture the distinguishing local features at different scales. The normalized multiresolution grouping (NMRG) helps the system to adapt the non-uniform density distribution of the 3D object point cloud from the input embedding. NMRG fuses features from the downsampling pyramid and upsampling pyramid of different scales and normalizes them. By leveraging the encoder and decoder structure of the transformer and incorporating T-net, POAT-Net is insensitive to the permutations of the point cloud and tolerates any initial rigid translation or rotation of the raw point cloud. The normalized NMRG and parallel offset-attention help POAT-Net improve the occluded object-detection rate. Ren et al. proposed a dynamic graph transformer 3D object-detection network (DGT-Det3D) [136]. The model consists of a dynamic graph transformer (DGT) and proposal-aware fusion (PAF) modules. The DGT module is a backbone network to encode long-range features and extracts spatial information. The proposed PAF module combines and enhances the spatial and point-wise semantic features for performance improvement. The model was trained and tested on the KITTI [52] and Waymo [90] datasets.

Theodose et al. [137] proposed a DL-based LiDAR resolution-agnostic model to minimize the effect of point cloud variation /distribution for 3D models. Two methods were proposed to improve the performance of a model for unknown data. The first one is increasing the data variability. The data variability increased by randomly discarding layers from the training. Each target was represented as a Gaussian function, and the corresponding loss function focused on the obstacle occupancy rather than regressing parameters as the other solution. The model has exclusively trained on the subset of KITTI [52] dataset and then evaluated on the nuScenes [63] and Pandaset [138] datasets. The model was trained only for car detection. Nagesh et al. [139] proposed an auxiliary network [121] to improve the localization accuracy of class imbalance on the nuScenes [63] dataset using the structure information of the 3D point cloud. The class-balanced grouping and sampling [140] was proposed to solve the classification loss problem due to class imbalance. The auxiliary network was combined with class-balanced grouping and sampling to improve the localization loss. The auxiliary network can be detached during the test time to reduce the computational burden. Wang et al. put forth a single-stage network, LSNet [141], which comprises a learned sampling (LS) module to sample important points. The LS sampling outperforms other techniques, such as farthest point sampling on the KITTI [52] dataset.

Hahner et al. [142] put forth a snowfall LiDAR simulation for 3D object detection. The snow particles were sampled for each LiDAR line in 2D space and used the induced geometry to modify each LiDAR beam measurement. Partially synthetic snow LiDAR data are generated and used these data to train several 3D networks, such as PV-RCNN [112], PointRCNN [14], SECOND [39], and PointPillars [41]. This technique is vital to simulate models when collecting data in such weather conditions is challenging.

Some methods use more than one LiDAR data representation to improve performance. Chen et al. developed Fast Point R-CNN [143], a two-stage hybrid 3D detection network using both voxel representations and raw point cloud data. In the first stage, VoxelRPN uses the voxel representations to make a few initial predictions. Each predicted bounding box in the first stage is projected to BEV. The second stage, RefinerNet, further improves the output of the first stage detection by directly processing the raw point cloud. The high-dimensional coordinate feature is fused with the convolutional feature to preserve accurate localization and context information. Shi et al. also proposed PV-RCNN [112] that uses both voxel representation and raw point clouds. The voxel-based part of the network encodes a multiscale representation of the features using sparse convolution. In contrast, the PointNet-based part of the network set abstraction to learn more discriminative features with small key-points. Each proposal introduces a multiscale ROI feature abstraction layer for points to preserve rich context information for accurate box refinement and confidence prediction. However, 3D convolution is still a bottleneck because of the high computational cost.

Likewise, Xu et al. presented Spg [144], an unsupervised domain adaptation model via semantic point generation. The model comprises three modules: the Voxel feature-encoding (VFE) module, the information propagation module, and the point generation module. The point cloud data are voxelized, and a prediction for each voxel, including occupied and empty, is generated. The VFE module from VoxelNet [9] aggregates points inside each voxel, and then the voxel features are stacked into pillars and projected onto a BEV feature space similar to PointPillars [41] and PV-RCNN [112]. The pillar features feed into the information propagation module for 2D convolutions operation. Then, the point generation module maps the pillar features to the corresponding voxels. These features are fed to the two detectors: Pointpillars [41] and PV-RCNN [112]. The model is evaluated on the Waymo open [90] and KITTI [52] datasets.

Table 1 gives the selected LiDAR-based 3D object-detection methods for the KITTI [52] dataset to show the performance improvement over time. The BEV and 3D KITTI evaluation benchmarks are commonly used to compare the performance of models in the KITTI dataset.

We recently addressed the challenges in 3D object detection, especially challenges related to processing point cloud data, LiDAR point encoding techniques, robust models, representative datasets, best fusion techniques, and others. We also indicated the possible research directions to solve the challenges in 3D object detection (Read [3,24] for more details). Different methods use different LiDAR encoding techniques and datasets. The comparison of LiDAR 3D object-detection methods based on the LiDAR encoding techniques, datasets, and publication years are summarized in Table 2 to show the performance improvement over time.

## 5. Conclusions

This survey presented state-of-the-art LiDAR-based 3D object detection for autonomous driving. The LiDAR feature-extraction methods and LiDAR encoding techniques were also summarized. The 3D coordinate systems are different for different sensors and datasets. Therefore, the commonly used 3D coordinate systems were reviewed. The stages of autonomous driving were also summarized. We categorized the 3D LiDAR perception systems methods based on the encoding technique as projection, voxel, and raw point cloud, with the pros and cons of each method. Generally, 3D object-detection methods show significant performance improvement for autonomous driving. However, several open issues exist in improving model speed and accuracy for real-time processing and level four and five driving. Some works, such as [145], proposed a computationally efficient algorithm for the quality of training and resource allocation, such as bandwidth and power allocation in multi-modal systems. We expect more works that efficiently train the complete system in autonomous driving.

## Figures and Tables

**Table 1:** BEV and 3D performance comparison (%) of selected LiDAR-based 3D object-detection methods on the KITTI [52] test benchmark. r40 shows the mAP is calculated for 40 recall points instead of 11. E stands for easy, M for moderate, and H for hard.

Methods	${\mathrm{AP}}_{\mathrm{BEV}}$									${\mathrm{AP}}_{3D}$								
	Car			Pedestrians			Cyclists			Car			Pedestrians			Cyclists		
	E	M	H	E	M	H	E	M	H	E	M	H	E	M	H	E	M	H
BirdNet [66]	75.5	50.8	50.0	26.1	21.4	20.0	39.0	27.2	25.5	14.8	13.4	12.0	14.3	11.8	10.6	18.4	12.4	11.9
BirdNet+ [67]	84.8	63.3	61.2	45.5	38.3	35.4	72.5	52.2	46.6	70.1	51.9	50.0	38.0	31.5	29.5	67.4	47.7	42.9
VoxelNet [9]	89.4	79.3	77.4	46.1	40.7	38.1	66.7	57.7	50.6	77.5	65.1	57.7	39.5	33.7	31.5	61.2	48.4	44.4
SECOND [39]	88.1	79.4	78.0	55.1	46.3	44.8	73.7	56.0	48.8	83.1	73.7	66.2	51.1	42.6	37.3	70.5	53.9	46.9
Fast point R-CNN [143]	88.0	86.1	78.2	-	-	-	-	-	-	84.3	75.7	67.4	-	-	-	-	-	-
PointPillars [41]	88.4	86.1	79.8	58.7	50.2	47.2	79.1	62.3	56.0	79.1	75.0	68.3	52.1	43.5	41.5	75.8	59.1	53.0
3DSSD [120]	88.4	79.6	74.6	-	-	-	-	-	-	-	-	-	-	-	-	-	-	-
SASSD [121]	88.8	79.8	74.2	-	-	-	-	-	-	-	-	-	-	-	-	-	-	-
CIA-SSD [123]	89.6	80.3	72.9	-	-	-	-	-	-	-	-	-	-	-	-	-	-	-
PIXOR++ [79]	89.4	83.7	78.0	-	-	-	-	-	-	-	-	-	-	-	-	-	-	-
TANet [88,141]	91.6	86.5	81.2	-	-	-	-	-	-	84.4	75.9	68.8	-	-	-	-	-	-
LSNet [141]	92.1	85.9	80.8	-	-	-	-	-	-	86.1	73.6	68.6	-	-	-	-	-	-
Associate-3Ddet [87]	91.4	88.1	83.0	-	-	-	-	-	-	86.0	77.4	70.5	-	-	-	-	-	-
HVNet [86] (r40)	92.8	88.8	83.4	54.8	48.9	46.3	84.0	71.2	63.7	-	-	-	-	-	-	-	-	-
Part-A2 [113]	91.7	87.8	84.6	-	-	-	-	-	-	87.81	78.49	73.51	-	-	-	-	-	-
PV-RCNN [112] (r40)	95.0	90.7	86.1	59.9	50.6	46.7	82.5	68.9	62.1	90.3	81.4	76.8	52.2	43.3	40.3	78.6	63.7	57.7
WCNN3D [59]	90.1	88.0	86.5	68.4	63.2	59.4	82.78	64.3	60.3	87.8	77.6	75.4	62.0	57.7	52.1	82.7	61.0	57.7
Point-GNN [124]	93.1	89.2	83.9	-	-	-	-	-	-	88.3	79.5	72.3	-	-	-	-	-	-
SE-SSD [131] (r40)	95.7	91.8	86.7	-	-	-	-	-	-	91.5	82.5	77.2	-	-	-	-	-	-

**Table 2:** Comparison of LiDAR-based 3D object-detection methods based on LiDAR encoding techniques, the dataset used, and year of publication. We categorized pillar representation in the volumetric encoding. Some methods use multiple datasets, but we report only datasets related to autonomous driving.

Method	LiDAR Encoding Technique	Dataset Used	Year of Publication
Yu et al. [64]	projection	KITTI	2017
BirdNet [66]	projection	KITTI	2017
Wirges et al. [65]	projection	KITTI	2018
PIXOR [78]	projection	KITTI	2018
Complex-YOLO [81]	projection	KITTI	2018
Birdnet+ [67]	projection	KITTI	2020
MVLidarNet [73]	projection	KITTI	2020
BirdNet+ [68]	projection	KITTI & Nuscenes	2021
YOLO3D [71]	projection	KITTI	2021
RAANet [76]	projection	KITTI & nuScenes	2021
Tian et al. [6]	Projection	nuScenes	2022
3DFCN [84]	voxel	KITTI	2017
VoxelNet [9]	voxel	KITTI	2018
SECOND [39]	voxel	KITTI	2018
F-PointNet [10]	voxel	KITTI & SUN RGB-D	2018
MVFP [11]	voxel	KITTI	2019
Frustum-ConvNet [99]	voxel	KITTI & SUN RGB-D	2019
Pointpillars [41]	pillar	KITTI	2019
McCrae and Zakhor [101]	pillar	KITTI	2020
Wang et al. [102]	pillar	Waymo	2020
HVNet [86]	voxel	KITTI	2020
Associate-3Ddet [87]	voxel	KITTI	2020
TANet [88]	voxel	KITTI	2020
Voxel R-CNN [89]	voxel	KITTI & Waymo	2021
SIENet [91]	voxel	KITTI	2021
Zhang et al. [105]	pillar	KITTI	2021
SMS-Net [92]	voxel	KITTI	2022
Sun et al. [93]	voxel	KITTI	2022
MA-MFFC [94]	voxel	KITTI	2022
SAT-GCN [95]	voxel	KITTI & Nuscenes	2022
Fan et al. [96]	voxel	Waymo	2022
Li et al. [91]	voxel	KITTI	2022
PDV [97]	voxel	KITTI & Waymo	2022
Fan et al. [103]	pillar	Waymo	2022
ASCNet [104]	pillar	KITTI	2022
PillarGrid [108]	pillar	synthetic data	2022
Lin et al. [110]	pillar	KITTI & CADC	2022
WCNN3D [59]	pillar	KITTI	2022
PointNet [12]	raw point cloud	ScanNet	2017
PointNet++ [13]	raw point cloud	ScanNet	2017
PointRCNN [14]	raw point cloud	KITTI	2019
STD [115]	raw point cloud	KITTI	2019
Part-A2 [113]	raw point cloud	KITTI	2020
3DSSD [120]	raw point cloud	KITTI & nuScenes	2020
SASSD [121]	raw point cloud	KITTI	2020
CIA-SSD [123]	raw point cloud	KITTI	2020
Point-GNN [124]	raw point cloud	KITTI	2020
Zhou et al. [125]	raw point cloud	KITTI	2020
Auxiliary network [121]	raw point cloud	nuScenes	2020
LSNet [141]	raw point cloud	KITTI	2021
Pyramid R-CNN [127]	raw point cloud	KITTI & Waymo	2021
ST3D [128]	raw point cloud	KITTI, Waymo, nuScenes & Lyft	2021
SE-SSD [131]	raw point cloud	KITTI	2021
Wang et al. [132]	raw point cloud	nuScenes & H3D	2021
POAT-Net [135]	raw point cloud	KITTI	2021
Theodose et al. [137]	raw point cloud	KITTI, nuScenes, & Pandaset	2021
DGT-Det3D [136]	raw point cloud	KITTI & Waymo	2022
Yu et al. [116]	raw point cloud	KITTI, ScanNetv2 & SUN RGB-D	2022
Hahner et al. [142]	raw point cloud	STF	2022
PointDistiller [134]	raw point cloud	KITTI	2022
Fast Point R-CNN [143]	voxel & raw point cloud	KITTI	2019
Pv-Rcnn [112]	voxel &raw point cloud	KITTI &Waymo	2020
Spg [144]	voxel & raw point cloud	KITTI &Waymo	2021

## Data Availability

Not applicable.

## Abbreviations

The following abbreviations are used in this manuscript:

BEV	Bird’s-Eye View
CNN	Convolutional Neural Network
DL	Deep Learning
GCN	Graph Convolution Network
IOU	Intersection Over Union
LiDAR	Light Detection and Ranging
mAP	Mean Average Precision
NMS	Non-maximal Suppression
RPN	Region Proposal Network
SOTA	State-of-the-art
VFE	Voxel Feature Encoding
WCNN3D	Wavelet Convolutional Neural Network for 3D Detection
YOLO	You Only Look Once
